# BAG6 contributes to glucose uptake by supporting the cell surface translocation of the glucose transporter GLUT4

**DOI:** 10.1242/bio.047324

**Published:** 2020-01-24

**Authors:** Setsuya Minami, Naoto Yokota, Hiroyuki Kawahara

**Affiliations:** Laboratory of Cell Biology and Biochemistry, Department of Biological Sciences, Tokyo Metropolitan University, Tokyo 192-0397, Japan

**Keywords:** Diabetes, Obesity, BAT3, Scythe, Rab8a, Membrane trafficking

## Abstract

Defective translocation of glucose transporter 4 (GLUT4) to the cell surface is a key feature of insulin resistance in type 2 diabetes. Therefore, elucidating the mechanism of GLUT4 translocation is of primary importance. The mammalian *Bag6/Bat3* gene has been suggested to be linked with potential obesity- and diabetes-associated loci, while its function in the control of glucose incorporation into the cytoplasm has not been investigated. In this study, we established a series of cell lines that stably expressed GLUT4 with three tandem repeats of the antigenic peptide inserted into its 1st extracellular loop. With these cell lines, we found that the depletion of endogenous BAG6 downregulated the cell surface expression of GLUT4, concomitant with the reduced incorporation of a glucose analog into the cells. Defective intracellular translocation of GLUT4 in BAG6-depleted cells is similar to the case observed for the depletion of Rab8a, an essential regulator of insulin-stimulated GLUT4 translocation. In addition, we observed that the assembly of syntaxin 6 into the endoplasmic reticulum membrane was slightly disturbed under BAG6 depletion. Given that Rab8a and syntaxin 6 are critical for GLUT4 translocation, we suggest that BAG6 may play multiple roles in the trafficking of glucose transporters to the cell surface.

This article has an associated First Person interview with the first author of the paper.

## Abstract (precis)

**Summary:** BAG6 is critical for the insulin-stimulated translocation of GLUT4 from its peri-nuclear storage compartments to the cell surface.

## INTRODUCTION

Glucose transport across the plasma membrane is a major rate-limiting step for glucose incorporation into the cell (Huang and Czech, 2007; Bogan, 2012). To increase cellular glucose uptake, insulin signaling promotes the translocation of specific glucose transporters to the plasma membrane (Huang and Czech, 2007; Bogan, 2012). Glucose transporter 4 (GLUT4), a predominant insulin-responsive glucose transporter, is sorted to the plasma membrane from peri-nuclear compartments, including GLUT4 storage vesicles (Rea and James, 1997; Huang and Czech, 2007; Li et al., 2009; Leto and Saltiel, 2012). In resting skeletal muscle and fat cells, the majority of GLUT4 is sequestered to the peri-nuclear compartments, preventing its translocation to the cell surface, in a dynamic equilibrium between these locations (Slot et al., 1991; Rea and James, 1997; Ploug et al., 1998; Ramm et al., 2000; Bryant et al., 2002; Eguez et al., 2005; Blot and McGraw, 2008). Insulin stimulates glucose uptake with the acute translocation of GLUT4 from peri-nuclear compartments to the cell surface (Bryant et al., 2002; Jaldin-Fincati et al., 2017; Aslamy and Thurmond, 2017). GLUT4 translocation can be abolished by wortmannin, an inhibitor of phosphoinositide 3-kinase (PI3K), an upstream kinase that phosphorylates Akt/protein kinase B (Summers et al., 1998; Ishikura et al., 2007; Randhawa et al., 2008; Ishikura and Klip, 2008; Sun et al., 2010; Sadacca et al., 2013; Lim et al., 2015; Li et al., 2017). Akt phosphorylation is necessary for the suppression of AS160, a GTPase-activating protein (GAP) for Rab8-family small GTPases (Eguez et al., 2005; Larance et al., 2005), and AS160 knockdown increases basal GLUT4 levels on the cell surface by activating a Rab8a-mediated membrane trafficking pathway (Eguez et al., 2005; Larance et al., 2005). According to these reports, dysfunction of Rab8-family small G proteins, such as Rab8a, Rab10 and Rab13, abrogates GLUT4 translocation (Sano et al., 2007; Sun et al., 2010; 2014; Chen et al., 2012; Sadacca et al., 2013; Bruno et al., 2016; Gulbranson et al., 2017). It was also reported that SNARE-mediated membrane fusion events determine GLUT4 localization (Bryant et al., 2002; Yu et al., 2013). These studies suggest that Rab8 family small G proteins and SNARE-mediated vesicle trafficking play critical roles in insulin-stimulated GLUT4 translocation.

Obesity and type 2 diabetes are characterized by insulin resistance (Zierath et al., 1996; Petersen and Shulman, 2006; Bogan, 2012; Foley and Klip, 2014; Aslamy and Thurmond, 2017). The defective translocation of GLUT4 in muscle cells, adipocytes and other cells is a key feature of insulin resistance (Klip et al., 1990; Zierath et al., 1996). GLUT4 translocation is deficient in most cases of type 2 diabetes; therefore, elucidating the regulatory mechanism of GLUT4 translocation is of primary importance. The *Bag6* gene [also called *Bat3* in humans (Banerji et al., 1990)] is linked to potential obesity loci, and differential alternative splicing of *Bag6* transcript is observed between overweight individuals with type 2 diabetes and lean individuals with normal glycemia (Kaminska et al., 2016). BAG6 protein possesses an intrinsic affinity for the exposed hydrophobicity of its client proteins in the cytosol, and escorts them to the degradation machinery (Kikukawa et al., 2005; Minami et al., 2010; Hessa et al., 2011; Wang et al., 2011; Lee and Ye, 2013; Suzuki and Kawahara, 2016; Tanaka et al., 2016; Guna and Hegde, 2018). BAG6 also recognizes the hydrophobic residues of Rab8a, which are specifically exposed in its GDP-bound form (Takahashi et al., 2019). This interaction stimulates the degradation of Rab8a (GDP), whose accumulation impairs Rab8a-mediated intracellular membrane trafficking.

Because Rab8a is a critical regulator for GLUT4 translocation (Ishikura et al., 2007; Randhawa et al., 2008; Ishikura and Klip, 2008; Sun et al., 2010; Sadacca et al., 2013; Li et al., 2017), we hypothesized that BAG6 might also have a function in the cell surface presentation of GLUT4. Therefore, the primary objective of this study was to investigate the possible participation of BAG6 in the insulin-stimulated cell surface translocation of GLUT4. In addition to its regulatory role in Rab8a degradation, BAG6 plays a partly redundant role in the biogenesis of tail-anchored (TA) proteins (Mariappan et al., 2010; Leznicki et al., 2010; Hegde and Keenan, 2011; Aviram et al., 2016; Casson et al., 2017; Haßdenteufel et al., 2017; Shao et al., 2017). Because several key SNARE components such as syntaxins are typical TA proteins (Hegde and Keenan, 2011; Casson et al., 2017), and because earlier studies highlighted the participation of syntaxin 6 (Stx6) in GLUT4 recycling (Perera et al., 2003; Shewan et al., 2003; Foley and Klip, 2014), we were interested in examining whether BAG6 depletion also affects Stx6 biogenesis.

In this study, we found that BAG6 knockdown induced the defective translocation of GLUT4 to the surface of the plasma membrane, concomitant with the reduced incorporation of a glucose analog into Chinese hamster ovary (CHO-K1) cells. This phenotype can be caused by the misregulation of Rab8a because the defective intracellular translocation of insulin-stimulated GLUT4 in Rab8a-depleted cells is similar to the case observed for BAG6 depletion. In addition, we found that the proper assembly of Stx6 into the endoplasmic reticulum (ER) membrane was moderately disturbed under BAG6 depletion. Given that Rab8a-family small GTPases and Stx6 are critical for GLUT4 translocation, we suggest that BAG6 may play multiple roles in glucose incorporation; thus, a deficiency of this triage factor might be a potential cause for some classes of obesity and type 2 diabetes.

## RESULTS

### BAG6 deficiency induces partial defects in glucose uptake in CHO cells

**Fig. 1.**
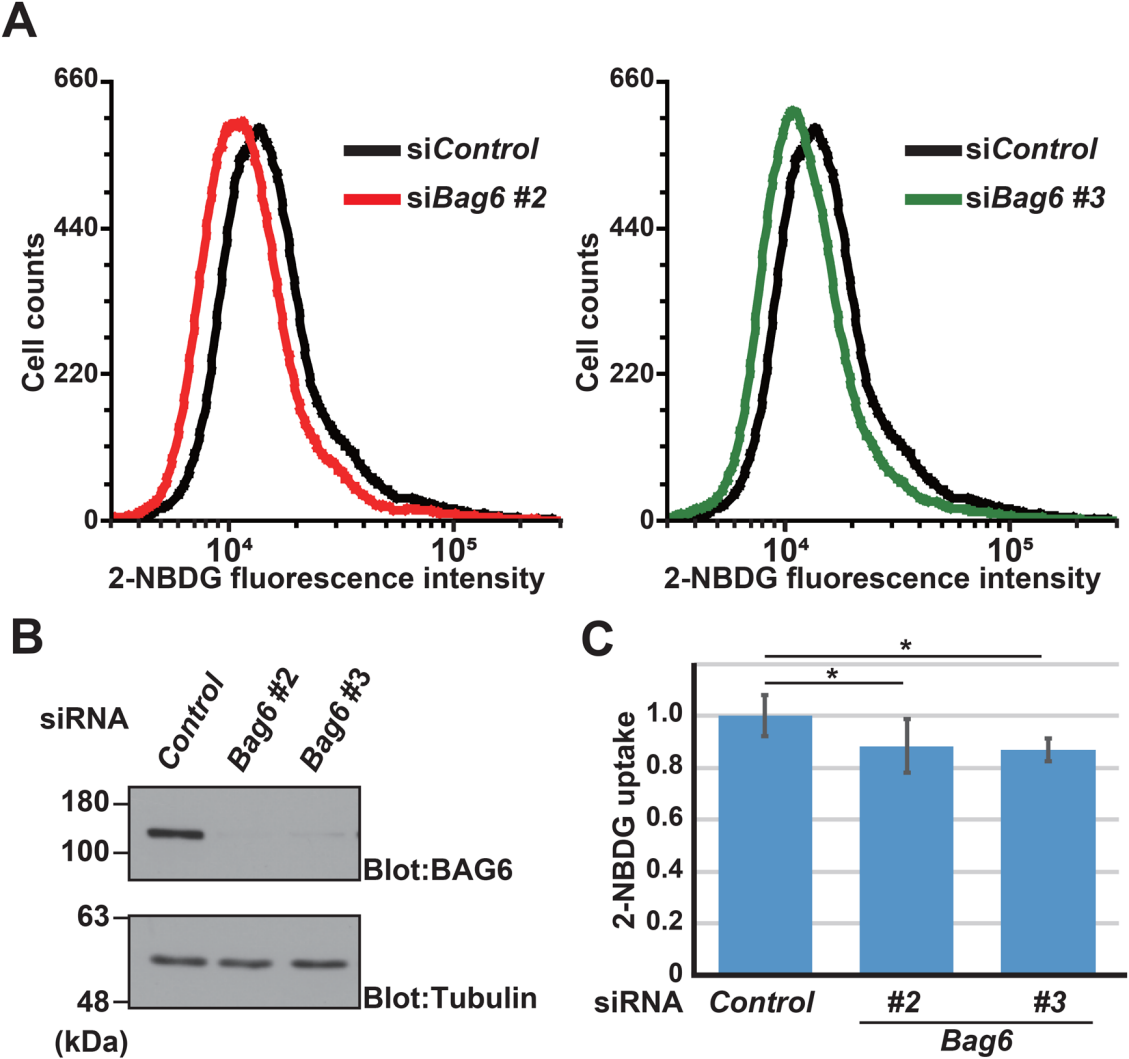
**BAG6 deficiency induces partial defects in glucose analog uptake in CHO cells.** (A) At 72 h after transfection with siRNA duplexes (5 nM each), the incorporation of the glucose analog 2-NBDG into CHO-K1 cells was quantified by live-cell flow cytometry analysis. Left panel: *Bag6* siRNA#2 (red line) and universal negative control siRNA (si*Control*, black line). Right panel: *Bag6* siRNA#3 (green line) and si*Control*. Insulin (1 μg/ml) was included in the medium. (B) Efficacy of BAG6 depletion with *Bag6* siRNA#2 and #3 duplexes in CHO-K1 cells. α-Tubulin was used as a loading control. (C) Quantification of 2-NBDG fluorescence with BAG6 depletion in the presence of 1 μg/ml insulin treatment. The value from si*Control* cells was defined as 1.0. The graph represents the mean±s.d. calculated from three independent biological replicates. Statistical significance was determined by Student's *t*-test. *P<0.05.

Rodent CHO-K1 cells reportedly possess glucose incorporation systems (Hasegawa et al., 1990; Johnson et al., 1998), and glucose transporters provide a route for the entry of glucose into CHO-K1 cells (Hasegawa et al., 1990; Kanai et al., 1993; Wei et al., 1998; Johnson et al., 1998; Bogan et al., 2001; Selvi et al., 2010). Using this cell line, we recently showed that BAG6 plays critical roles in the appropriate trafficking of Golgi/endosomal proteins (Takahashi et al., 2019). During the course of our study, we noticed that the function of BAG6 might be linked with glucose uptake events in this cell line. Flow cytometry analyses suggested that the incorporation of 2-NBDG, a fluorescently labeled deoxyglucose analog, into CHO-K1 cells was compromised modestly by the depletion of endogenous BAG6 (Fig. 1A). To avoid possible off-target effects of small interfering RNA (siRNA), we examined two independent double-stranded RNA constructs (*Bag6* siRNA#2 and #3, see Materials and Methods for their respective target sequences). Western blot analysis with an anti-BAG6 antibody confirmed that both *Bag6* siRNA#2 and #3 constructs efficiently depleted endogenous BAG6 protein from CHO-K1 cells (Fig. 1B). Both of these siRNA constructs similarly suppressed the incorporation of 2-NBDG compared with the case with the universal negative control siRNA construct (si*Control*) (Fig. 1A). Quantification of the mean value of 2-NBDG fluorescence confirmed that BAG6 depletion downregulated 2-NBDG uptake significantly (Fig. 1C). Although the effect of BAG6 depletion on the reduction of mean 2-NBDG fluorescence was rather limited, we hypothesized that this partial reduction of 2-NBDG uptake induced by BAG6 depletion might be attributed to defects in the cell surface expression of glucose transporters.

### Establishment of transgenic cell lines expressing GLUT4 with Flag-tags in the extracellular domain

**Fig. 2.**
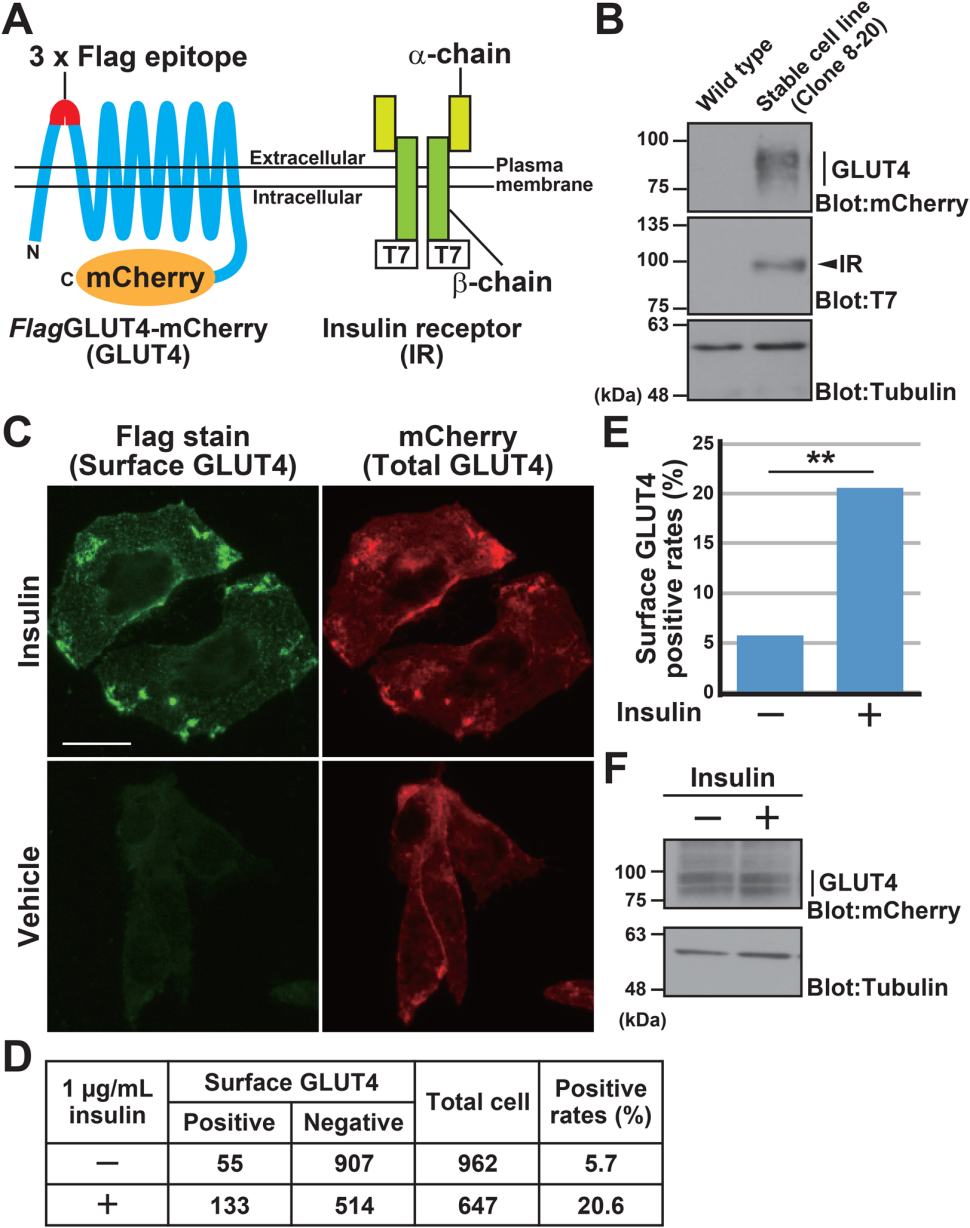
**Transgenic cell lines with Flag-tag inserted in the extracellular domain of GLUT4.** (A) Schematic of the Flag-inserted GLUT4-mCherry (left panel) and T7-tagged IR (right panel) proteins used in this study. Both of these gene products were stably co-expressed in CHO-K1 cells. A triple repeat of the Flag epitope tag (3×Flag) was inserted into the first extracellular loop of GLUT4 between Pro^66^ and Gly^67^ (Kanai et al., 1993). With this inserted epitope, cell surface GLUT4 can be detected on non-permeabilized cells using an anti-Flag M2 antibody. The C-terminus of GLUT4 was fused with mCherry to quantify the total level of GLUT4 expression. The C-terminus of the IR β-chain was fused with a 3×T7 epitope tag (IR-T7). (B) Stable expression of Flag-inserted GLUT4-mCherry protein and IR-T7 protein in a transgenic CHO-K1 cell line (clone 8-20) was verified by western blot analysis. α-Tubulin was used as a loading control. The non-transfected parental CHO-K1 cell line was used as a negative control (wild-type). (C) Insulin stimulates the cell surface expression of *Flag*GLUT4-mCherry. Plasma membrane exposed (shown as green) or total (shown as red) GLUT4 protein levels were examined with (Insulin) or without (Vehicle) insulin treatment (1 μg/ml). In order to detect cell surface GLUT4 exclusively, the plasma membrane was intentionally left non-permeabilized. Transgenic cell line clone 8-20 was used in this experiment. Scale bar: 20 µm. A Keyence BZ-X700 fluorescence microscope was used for observations. (D,E) Cell surface Flag-positive cells were counted with (+) or without (−) insulin. *n*=962 cells for control, and *n*=647 cells for insulin treatment. Statistical significance in E was determined by a chi-square test. **P<0.01 compared with control. (F) Anti-mCherry immunoblot analysis showed that insulin treatment did not influence the total expression level of *Flag*GLUT4-mCherry fusion protein. α-Tubulin was used as a loading control.

Plasma membrane-localized glucose transporters are responsible for extracellular glucose incorporation into the cytoplasm (Huang and Czech, 2007; Bogan, 2012). Since *Bag6* has been linked to potential obesity- and type 2 diabetes-associated loci (Kaminska et al., 2016; Carayol et al., 2017), and since BAG6 regulates the stability of the intrinsically unstable Rab8a-GDP-bound form (Takahashi et al., 2019), a small G protein that has been shown to be critical for insulin-stimulated GLUT4 sorting to the plasma membrane (Ishikura et al., 2007; Randhawa et al., 2008; Ishikura and Klip, 2008; Sun et al., 2010; Sadacca et al., 2013; Li et al., 2017), we were interested in examining the cell surface expression of GLUT4 (Rea and James, 1997; Li et al., 2009; Leto and Saltiel, 2012). To investigate this point, we established a series of CHO-K1 cell lines that were stably co-transfected with *Glut4* as well as insulin receptor (*IR*) genes (Fig. 2A,B). According to a previously established method to detect the cell surface expression of GLUT4 (Kanai et al., 1993; Ueyama et al., 1999; Ishikura et al., 2007), we inserted antigenic peptides (three tandem repeats of the Flag-tag sequence were inserted instead of the original single Myc-tag to increase detection sensitivity) into the 1st extracellular loop of GLUT4 (between Pro^66^ and Gly^67^, Fig. 2A). In addition, the mCherry tag was fused to the C-terminus of GLUT4 (designated as *Flag*GLUT4-mCherry) to monitor the total level and intracellular localization of GLUT4 (Fig. 2A). Using one of these transgenic cell lines (clone 8-20), we confirmed that the number of cell surface Flag-positive cells was increased in response to insulin (Fig. 2C). Indeed, 20.6% of cells were positive for cell surface Flag-inserted GLUT4 expression under insulin exposure, while only 5.7% of non-treated cells were positive (Fig. 2D,E), although the total amount of GLUT4 protein was not significantly changed by insulin treatment (Fig. 2F).

### BAG6 deficiency induces defects in the cell surface expression of GLUT4

**Fig. 3.**
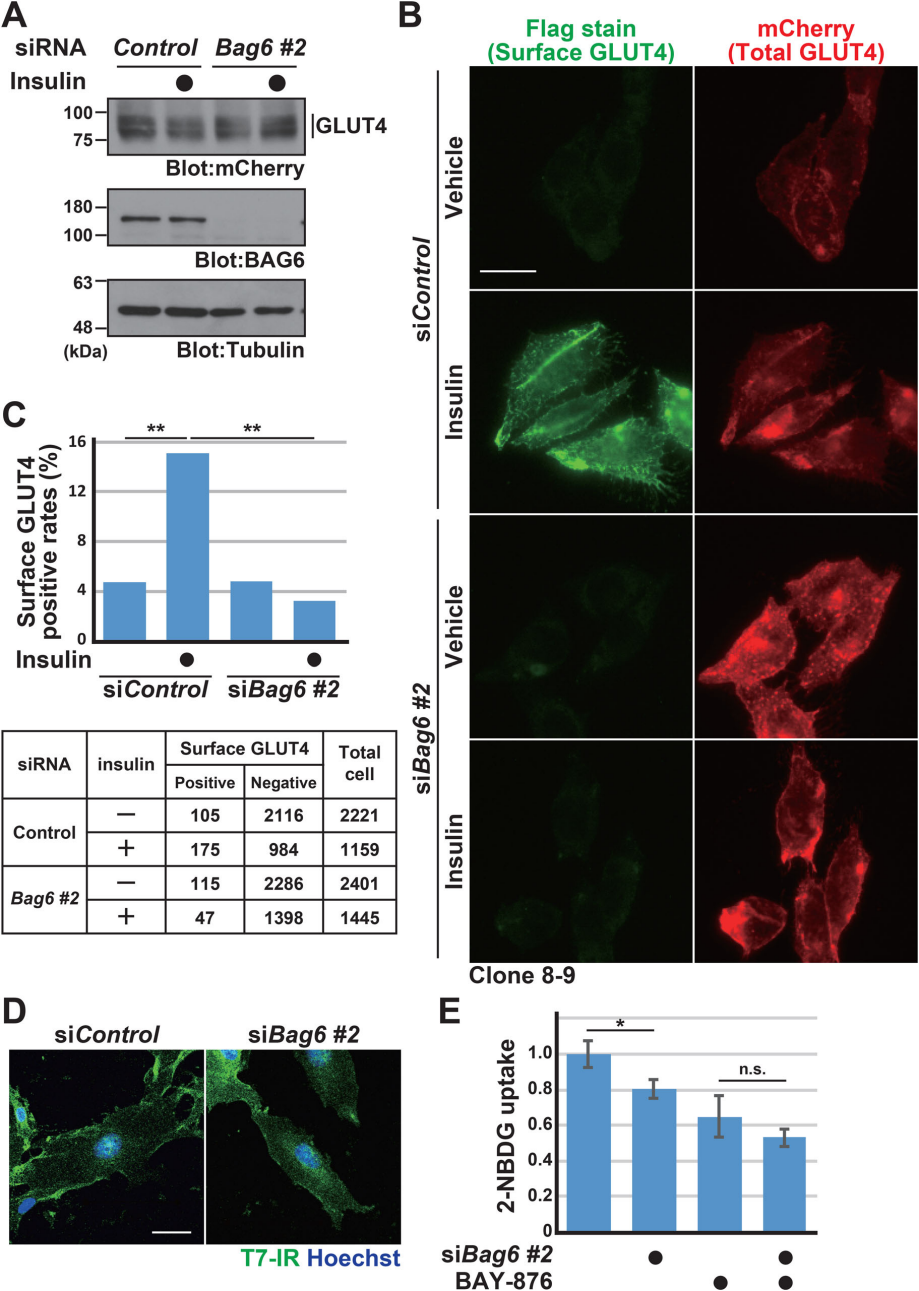
**BAG6 deficiency induces defects in the cell surface expression of GLUT4.** (A) Efficacy of BAG6 knockdown and the expression levels of *Flag*GLUT4-mCherry protein in CHO-K1 cells. Note that BAG6 depletion did not influence the total expression level of GLUT4. (B) At 72 h after transfection with siRNA duplexes (5 nM each) for *Bag6* siRNA#2 (lower panels) or control (upper panels) into a transgenic CHO-K1 cell line (clone 8-9), plasma membrane-exposed (Flag-signals on non-permeabilized cells are shown as green in the left panels) or total (mCherry signals are shown as red in the right panels) GLUT4 protein levels were observed with insulin treatment. Keyence BZ-X700 fluorescence microscope was used for observations. Scale bar: 20 µm. See also Fig. S1. (C) Cell surface Flag-positive cells were counted under the respective conditions and the positive rates are plotted as a bar graph. Transgenic cell line clone 8-20 was used in this experiment. The quantified data represent positive rates. *n*=1159 cells for control siRNA, *n*=1445 cells for *Bag6* siRNA#2. Statistical significance was determined by a chi-square test. **P<0.01 compared with control. (D) Cellular distribution of IR-T7 (green) in control or BAG6 knockdown cells. T7-immunosignals were detected under a cell membrane-permeabilized condition. Nuclear DNA was stained with Hoechst 33342 (shown as blue). Scale bar: 20 µm. (E) The incorporation of 2-NBDG into a transgenic CHO-K1 cell line (clone 8-20) was quantified as in Fig. 1. Insulin (1 μg/ml) was included in the medium. BAY-876 (150 nM) was included as a glucose transporter inhibitor as indicated. The value from control cells was defined as 1.0. The graph represents the mean±s.d. calculated from three independent biological replicates. Statistical significance was determined by Student's *t*-test. *P<0.05; n.s., not significant.

To examine whether BAG6 regulates GLUT4, we evaluated the effect of BAG6 knockdown on the cell surface expression of *Flag*GLUT4-mCherry in the transgenic cell lines established in the previous section. The efficacy of BAG6 depletion in the GLUT4 transgenic cell line was verified by western blot analysis (Fig. 3A; BAG6 blot). We also confirmed that BAG6 knockdown did not affect the total expression level of GLUT4 protein (Fig. 3A; mCherry blot). Anti-Flag M2 immunofluorescent microscopy with non-permeabilized cells revealed that a portion of insulin-stimulated transgenic cells showed the clear cell surface expression of Flag-inserted GLUT4 (Fig. 3B; green signals in the si*Control* panel). In contrast, the depletion of endogenous BAG6 greatly downregulated the cell surface expression of Flag-inserted GLUT4, even in the presence of insulin (Fig. 3B; green signals in the *Bag6* siRNA#2 panel). We confirmed this observation with two independently isolated transgenic cell lines (clone 8-9 for Fig. 3B, and clone 8-20 for Fig. S1). In control knockdown cells with insulin treatment, 15.1% of cells were positive for cell surface GLUT4, while only 3.3% of BAG6 knockdown cells were positive (Fig. 3C). A chi-square test suggested that the effect of BAG6 depletion on the number of cell surface GLUT4-positive cells was statistically significant (Fig. 3C). In spite of the clear reduction of cell surface GLUT4, we confirmed that the cell surface expression of T7-tagged IR (IR-T7) was normal in BAG6-suppressed cells (Fig. 3D). The incorporation of the glucose analog 2-NBDG into a transgenic CHO-K1 cell line in the presence of insulin was also compromised by either the depletion of endogenous BAG6 or treatment with BAY-876, a selective inhibitor of glucose transporter GLUT1 (Fig. 3E).

**Fig. 4.**
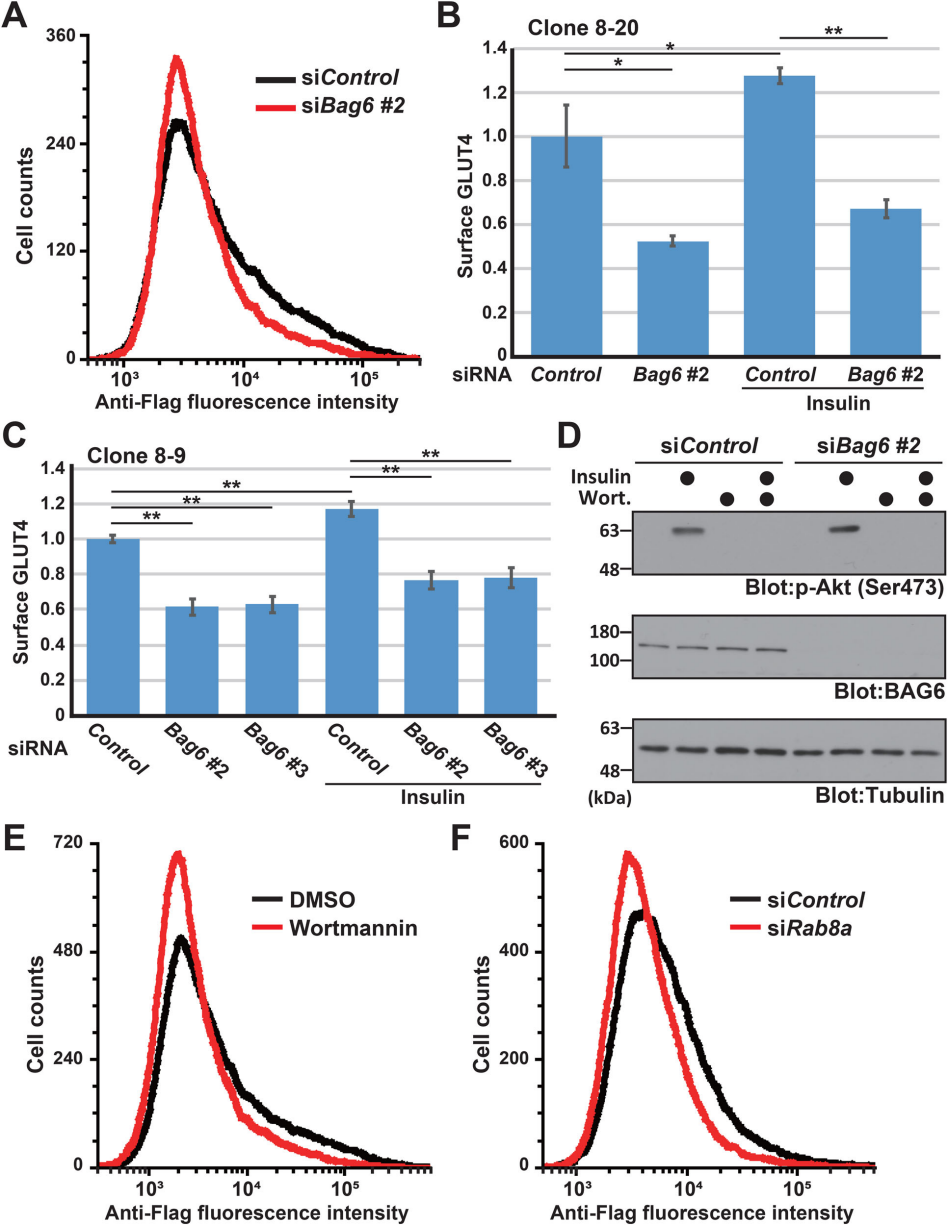
**Live-cell flow cytometry analysis indicates that BAG6 is necessary for the cell surface expression of GLUT4.** (A) BAG6 depletion downregulates the cell surface expression of GLUT4. Live-cell flow cytometry analysis of a non-permeabilized CHO-K1 cell line (clone 8-20) with an anti-Flag M2 antibody. The flow cytometry patterns of negative control and *Bag6* siRNA#2 are indicated as black and red lines, respectively. BAG6 knockdown was performed with three independent siRNA duplexes as described in the Materials and Methods, which all gave similar results (see Fig. S2A,B). Representative results for *Bag6* siRNA#2 are shown. Insulin (1 μg/ml) was included in the culture medium. The data were obtained by logarithmic scale analysis. (B) Quantitative evaluations of the flow cytometric fluorescence intensity of cell surface GLUT4 (determined by anti-Flag immunosignals) of transgenic CHO-K1 cells (clone 8-20). The data shown are the calculated cell surface GLUT4 ratio normalized by the intensity of control siRNA cells without insulin. See also Fig. S2C,D. (C) Quantitative evaluations of the fluorescence intensity of cell surface GLUT4 of a different transgenic CHO-K1 cell line (clone 8-9) that was isolated independently to clone 8-20. (D) Akt phosphorylation at Ser^473^ was examined using an anti-phospho Akt antibody. The results suggest that insulin stimulates Akt phosphorylation in a PI3K-dependent manner, and BAG6 depletion did not affect Akt phosphorylation. (E,F) Live cell flow cytometry analysis as in A. To block PI3K/Akt-dependent GLUT4 translocation, 100 nM wortmannin was added to the culture medium at 10 min before insulin stimulation (clone 8-20). The flow cytometry patterns are indicated as negative control (E,F, black lines), wortmannin treatment (E, red line), and *Rab8a* siRNA (F, red line). All the data were confirmed by at least three independent biological replicates. Statistical significance was determined by Student's *t*-test. *P<0.05, **P<0.01.

Flow cytometry quantification of cell surface Flag-inserted GLUT4 further supported this conclusion. Under insulin treatment, the number of cell surface Flag-positive cells was reproducibly impaired by three independent siRNA constructs for *Bag6*, i.e., *Bag6* siRNA#2 (Fig. 4A,B), *Bag6* siRNA#3 (Fig. S2A,C,E), and *Bag6* siRNA#5 (Fig. S2B,D,E). For *Bag6* siRNA#2, BAG6 depletion significantly reduced the amount of cell surface GLUT4 by more than 48% compared with control knockdown (Fig. 4B). The other two independent siRNA constructs for *Bag6* were equally effective at downregulating the cell surface expression of GLUT4 (Fig. S2C,D,E), thus reducing the possibility of adventitious off-target effects. Furthermore, we confirmed that another independently isolated *Flag*GLUT4-mCherry transgenic cell line (clone 8-9) also showed a nearly identical response to *Bag6* siRNA#2 and siRNA#3 (Fig. 4C), supporting the reproducibility of these observations. All of these results support the notion that BAG6 possesses a critical role in the cell surface translocation of GLUT4 in transgenic CHO-K1 cells. It was unexpected for us to see the partial effect of BAG6 knockdown on the amount of cell surface GLUT4 even under the basal state (without insulin).

Because insulin-dependent GLUT4 translocation is regulated by Akt kinase (Ishikura et al., 2007; Randhawa et al., 2008; Ishikura and Klip, 2008; Sun et al., 2010; Sadacca et al., 2013; Lim et al., 2015; Li et al., 2017), we examined whether BAG6 knockdown affected the insulin-dependent activation of Akt. We confirmed that insulin-dependent Akt phosphorylation was not perturbed in BAG6 knockdown cells (Fig. 4D), suggesting that intracellular insulin signaling including the function of IR is normal in these cells. Treatment with wortmannin, an inhibitor of PI3K, which is an upstream regulator of the Akt-Rab8a signaling pathway (Fig. S2F), abolished Akt phosphorylation (Fig. 4D). Wortmannin treatment also reduced the insulin-dependent cell surface expression of GLUT4 (Fig. 4E), similar to the case for Rab8a knockdown (Fig. 4F), as reported previously (Summers et al., 1998; Eguez et al., 2005; Ishikura and Klip, 2008; Sadacca et al., 2013; Lim et al., 2015; Li et al., 2017). Flow cytometry of wortmannin-treated and Rab8a-depleted cells showed a nearly indistinguishable cell surface GLUT4 expression pattern to that of BAG6-depleted cells (compare Fig. 4A,E,F). These observations suggest that BAG6 and Rab8a function in a largely overlapping pathway.

### BAG6 is required for the insulin-stimulated relocation of intracellular GLUT4

Previously, we reported that the intracellular localization of Patched1, a GLUT4-related 12 membrane-passed sterol transporter protein, is disturbed by Rab8a depletion (Takahashi et al., 2019). Furthermore, the endosomal localization of Patched1 in HeLa cells is impaired in BAG6-depleted cells (Takahashi et al., 2019). These results suggest that the intracellular localization of Patched1 might be controlled by a BAG6- and Rab8a-dependent pathway. Because Rab8a is also critical for the insulin-stimulated translocation of GLUT4 from specialized peri-nuclear compartments to the plasma membrane (Ishikura et al., 2007; Sun et al., 2010; Li et al., 2017), we examined whether BAG6 knockdown affected the intracellular localization of GLUT4 in the presence or absence of insulin stimulation.

**Fig. 5.**
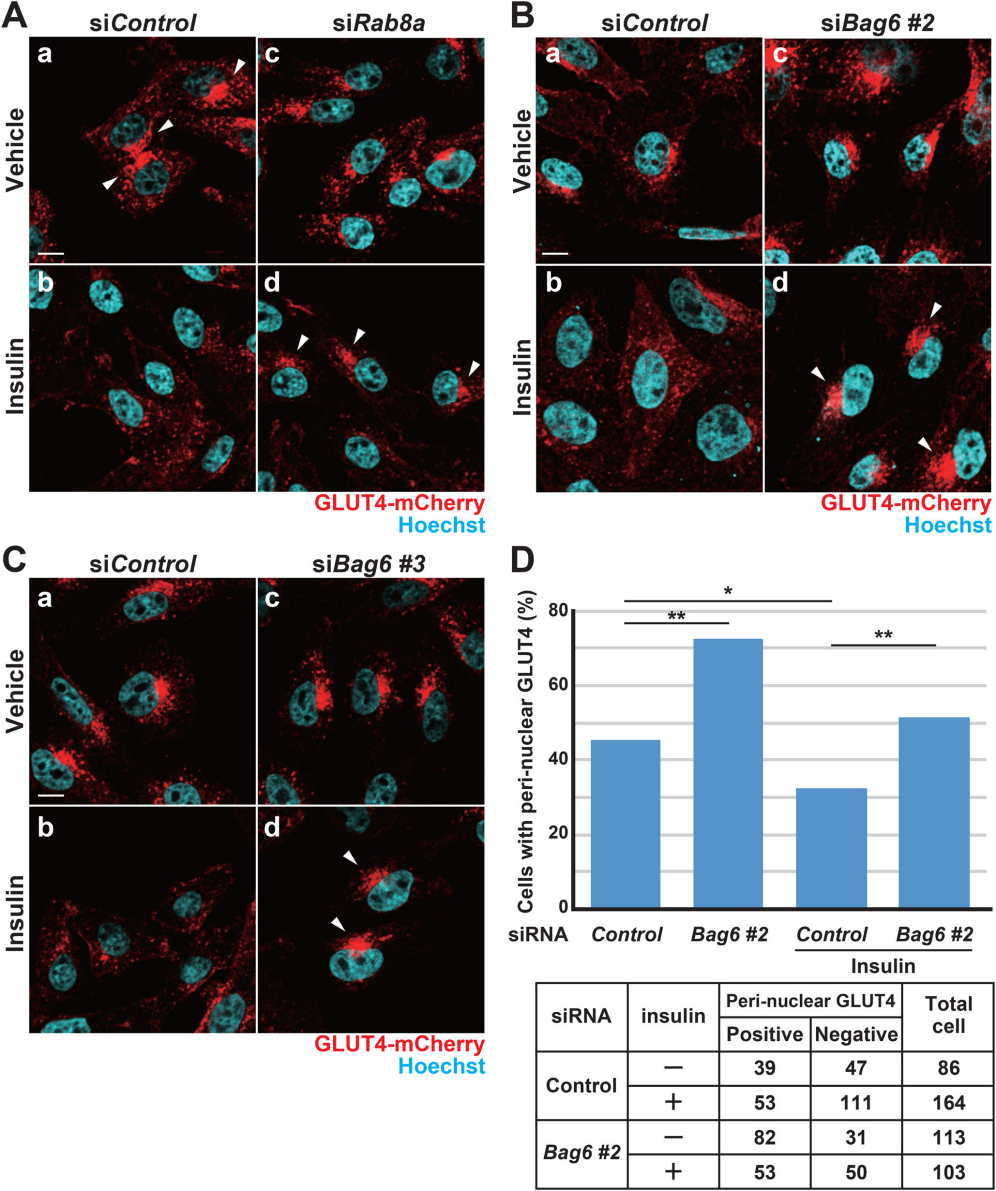
**Defective intracellular distribution of insulin-stimulated GLUT4 in BAG6-suppressed cells.** (A–C) Intracellular localization of *Flag*GLUT4-mCherry (red) in the presence (Insulin) or absence (Vehicle) of insulin stimulation with or without *Rab8a* siRNA (A), *Bag6* siRNA#2 (B) and *Bag6* siRNA#3 (C). Fluorescent mCherry-derived signals were detected using a laser scanning confocal microscopy system (LSM710). Note that this experiment used a different transgenic cell line (clone 51-25) because we noticed that the endogenous expression of IR in CHO-K1 cells is sufficient for insulin responsiveness and that IR transfection is dispensable for insulin-dependent Akt phosphorylation. We observed similar results with clone 8-20. Nuclei were stained with Hoechst 33342 (shown as blue). Peri-nuclear-localized GLUT4 signals are indicated by white arrowheads. Scale bars: 10 µm. (D) Quantification of the number of cells with the peri-nuclear localization of GLUT4 with or without *Bag6* siRNA#2. Statistical significance was determined by chi-square test. *P<0.05, **P<0.01.

Confocal microscopy observations suggested that *Flag*GLUT4-mCherry was localized to specific peri-nuclear compartments in the majority of control cells under the basal condition (Fig. 5A-a, indicated by white arrowheads). In contrast, the GLUT4 signal was diffused throughout the cytosol with insulin stimulation (Fig. 5A-b), suggesting that the active intracellular translocation of GLUT4 protein was stimulated by insulin, as previously reported (Rea and James, 1997; Ploug et al., 1998; Ramm et al., 2000; Randhawa et al., 2008; Fujita et al., 2010; Boguslavsky et al., 2012). In the case of Rab8a-depleted cells, the insulin-stimulated diffusion of GLUT4 from the peri-nuclear compartments was severely inhibited (Fig. 5A-d, white arrowheads). Similar to the case for Rab8a knockdown, the *Flag*GLUT4-mCherry signals under insulin stimulation remained around the peri-nuclear compartments following *Bag6* depletion with siRNA#2 (Fig. 5B-d, white arrowheads). The defective response of GLUT4 translocation to insulin was also reproduced with *Bag6* siRNA#3 (Fig. 5C-d, white arrowheads). The peri-nuclear localization of GLUT4 under the basal condition was not obviously disturbed by BAG6 or Rab8a knockdown (Fig. 5A-c,B-c,C-c). Quantification of GLUT4 localization supported the statistical significance of the enhanced peri-nuclear accumulation of GLUT4 in BAG6-suppressed cells in the presence of insulin (Fig. 5D). These observations suggest that endogenous BAG6 protein is necessary, similar to Rab8a, for the insulin-stimulated translocation of intracellular GLUT4 from peri-nuclear compartments in CHO-K1 transgenic cells.

### BAG6 has a partial role in the membrane integration of Stx6

**Fig. 6.**
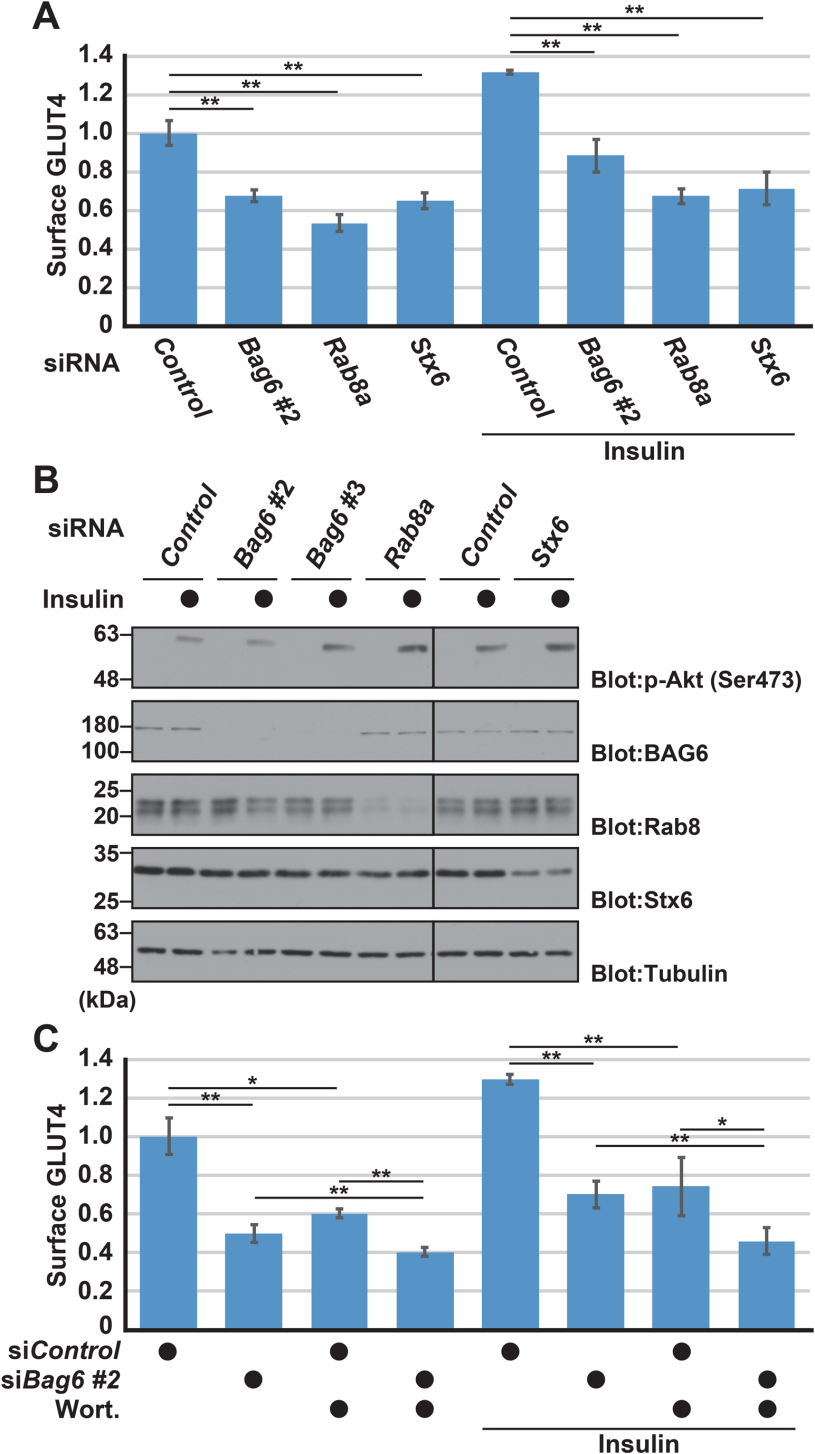
**Cell surface expression of GLUT4 is downregulated by BAG6, Rab8a and Stx6 depletion.** (A) Flow cytometry analysis of GLUT4 with BAG6, Rab8a and Stx6 knockdown. The intensity of cell surface GLUT4 signals is indicated as relative values to the signal of the basal (no insulin) condition. (B) Insulin-stimulated Akt phosphorylation at Ser^473^ was not perturbed by BAG6, Rab8a and Stx6 knockdown. (C) Flow cytometry quantification of cell surface GLUT4 expression with BAG6 knockdown in the presence of insulin (clone 8-20). To block PI3K/Akt-dependent GLUT4 translocation, 100 nM wortmannin (Wort.) was added to the culture medium at 10 min before insulin stimulation. All data were confirmed by at least three independent biological replicates. Statistical significance was determined by Student's *t*-test. *P<0.05, **P<0.01.

Rab8a, a Golgi/endosomal small G protein, and Stx6, a Golgi apparatus t-SNARE protein, are critical for the insulin-stimulated cell surface expression of GLUT4 (Perera et al., 2003; Shewan et al., 2003; Foley and Klip, 2014; Li et al., 2017). In accordance with these reports, we found that Rab8a knockdown (and wortmannin treatment) significantly reduced the cell surface expression of GLUT4 in our transgenic cell lines, similar to the case for BAG6 knockdown (Figs 4F and 6A). We confirmed that insulin-stimulated Akt activation was normal in these knockdown cells (Fig. 6B). However, we noticed that a non-negligible sensitivity to BAG6 knockdown was evident even when the Akt-mediated signaling pathway was suppressed using a PI3K inhibitor (Fig. 6C, compare *control* and *Bag6* siRNA in the presence of wortmannin). This observation suggests that a PI3K-/Akt-/Rab8a-independent pathway might also have a role in BAG6-sensitive GLUT4 translocation, although a major part of the defects induced by BAG6 depletion can be attributed to a PI3K-dependent pathway.

Since BAG6 was also suggested as a component of the TA protein membrane assembly machinery (Mariappan et al., 2010; Leznicki et al., 2010; Hegde and Keenan, 2011; Casson et al., 2017; Shao et al., 2017) and since Stx6 is a TA protein whose dysfunction affects the cell surface expression of GLUT4 (Fig. 6A), we suspected that the observed GLUT4 trafficking defects under BAG6 depletion might partly be due to a failure in Stx6 function, in addition to the misregulation of Rab8a (Takahashi et al., 2019). Therefore, we examined whether Stx6 was synthesized properly under BAG6 depletion.

**Fig. 7.**
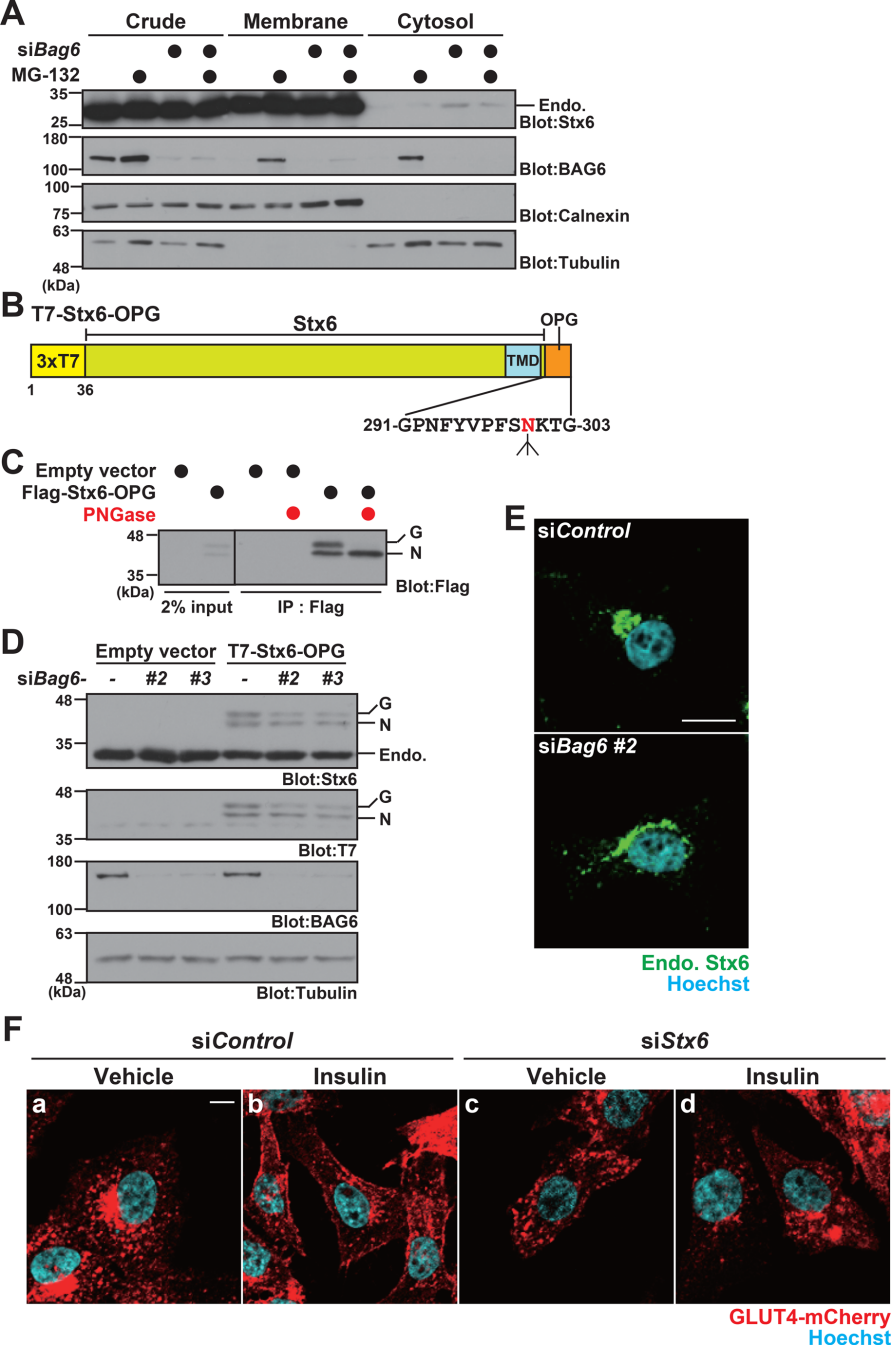
**BAG6 has a partial role in Stx6 biogenesis.** (A) CHO-K1 cells transfected with 5 nM *Bag6* siRNA#2 duplex or control siRNA were fractionated into cytosolic and membrane-associated/insoluble fractions and were probed with an anti-Stx6 antibody to detect the cytoplasmic defective form of endogenous Stx6. The BAG6 blot indicates the depletion of BAG6 protein by its siRNA. α-Tubulin was used as a cytoplasmic marker and calnexin was used as a membrane fraction marker. (B) Schematic of the C-terminal OPG-tagged and N-terminal T7- (or Flag-) tagged Stx6 used in this study. The amino acid sequences of the OPG tag are indicated with the N-glycosylation site at Asn^300^. TMD indicates the transmembrane domain. (C) C-terminally OPG-tagged Stx6 was glycosylated. Flag-Stx6-OPG protein was expressed in CHO-K1 cells and immunoprecipitated with an anti-Flag antibody. The precipitates were incubated with or without five units of the de-glycosylation enzyme PNGase F and subjected to western blot analysis with an anti-Flag antibody. Glycosylated (indicated as G) and non-glycosylated (indicated as N) signals are indicated. (D) Glycosylation of OPG-tagged Stx6 was reduced modestly by BAG6 knockdown. T7-tagged Stx6-OPG was expressed in CHO-K1 cells with or without *Bag6* siRNA (duplexes #2 and #3), and probed with anti-Stx6 and an anti-T7 antibodies. Low-mobility glycosylated (G) and high-mobility non-glycosylated (N) signals of Stx6-OPG are indicated. α-Tubulin was used as a loading control. Endo. indicates a specific signal for endogenous Stx6 protein. (E) Abnormal distribution of endogenous Stx6 (Endo. Stx6, shown as green) in BAG6-depleted CHO-K1 cells (with *Bag6* siRNA#2). Fluorescent signals were detected using a laser scanning confocal microscopy system (LSM710). Nuclei were stained with Hoechst 33342 (shown as blue). Scale bar: 10 µm. (F) Intracellular localization of *Flag*GLUT4-mCherry (red) in the presence (Insulin, b,d) or absence (Vehicle, a,c) of insulin stimulation with (c,d) or without (a,b) *Stx6* siRNA. Fluorescent mCherry-derived signals were detected as in Fig. 5. Scale bar: 10 µm.

To examine the assembly of Stx6 into the ER membrane, we performed a cell fractionation assay. We confirmed that the majority of endogenous Stx6 was present in the membrane fraction in control and BAG6 knockdown cells (Fig. 7A, membrane fractions), suggesting that a major portion of Stx6 in CHO-K1 cells was successfully assembled into the membrane fraction, even in the absence of BAG6. However, we found that a small portion of endogenous Stx6 was present in the cytosolic soluble fraction in BAG6-suppressed cells (Fig. 7A, cytosolic fractions). This observation implies a partial defect in the ER assembly of Stx6 in BAG6-suppressed cells.

To investigate further whether a portion of Stx6 failed to incorporate into the ER membrane, we prepared an expression system for Stx6 with a C-terminal N-glycosylation (OPG) tag (Fig. 7B), which is modified with N-linked glycans by ER-resident glycosylation enzymes only when it is successfully inserted into the ER lumen (Fig. 7B). We confirmed that Stx6-OPG was detected as doublet bands due to its glycosylation (Fig. 7C), and the upper signals (indicated as G) were shifted down by treatment with PNGase F, a de-glycosylation enzyme (Fig. 7C, indicated as N), suggesting that a part of Stx6-OPG was integrated properly into the ER membrane. With this experimental system, we analyzed the glycosylation state of Stx6-OPG in BAG6 knockdown cells. As shown in Fig. 7D, the intensity of the N-glycosylated (i.e. membrane-integrated) bands of OPG-tagged Stx6 was reduced modestly in BAG6-suppressed cells (Fig. 7D, indicated as G), suggesting that the efficacy of Stx6 biogenesis was partly dependent on BAG6. This was in contrast to the case of other major TA proteins, such as OPG-tagged RAMP4, BNIP1, and Sec61, whose membrane assembly efficiency was scarcely affected by BAG6 knockdown in HeLa cells (Fig. S3). We concluded that Stx6 assembly into the ER membrane was selectively, but only moderately, perturbed by BAG6 depletion, similar to the case of Stx5 following the depletion of TRC40, a BAG6-associated protein (Casson et al., 2017). In addition to the compromised assembly of Stx6 into the ER membrane, BAG6 knockdown also induced the defective localization of endogenous Stx6 (Fig. 7E). Finally, we examined whether Stx6 dysfunction affected the intracellular localization of GLUT4. As shown in Fig. 7F, the peri-nuclear localization of GLUT4 was perturbed by Stx6 knockdown, not only in the presence of insulin but also under the basal state (in the absence of insulin stimulation). Because the phenotype observed in Stx6-depleted cells was found to be partly distinct from that for BAG6 knockdown (compare Figs 5B-c and 7F-c in the absence of insulin, and Figs 5B-d and 7F-d in the presence of insulin, respectively), we suggest that the defective synthesis of Stx6 might contribute, only in part, to the reduced cell surface expression of GLUT4 in BAG6-suppressed cells.

## DISCUSSION

**Fig. 8.**
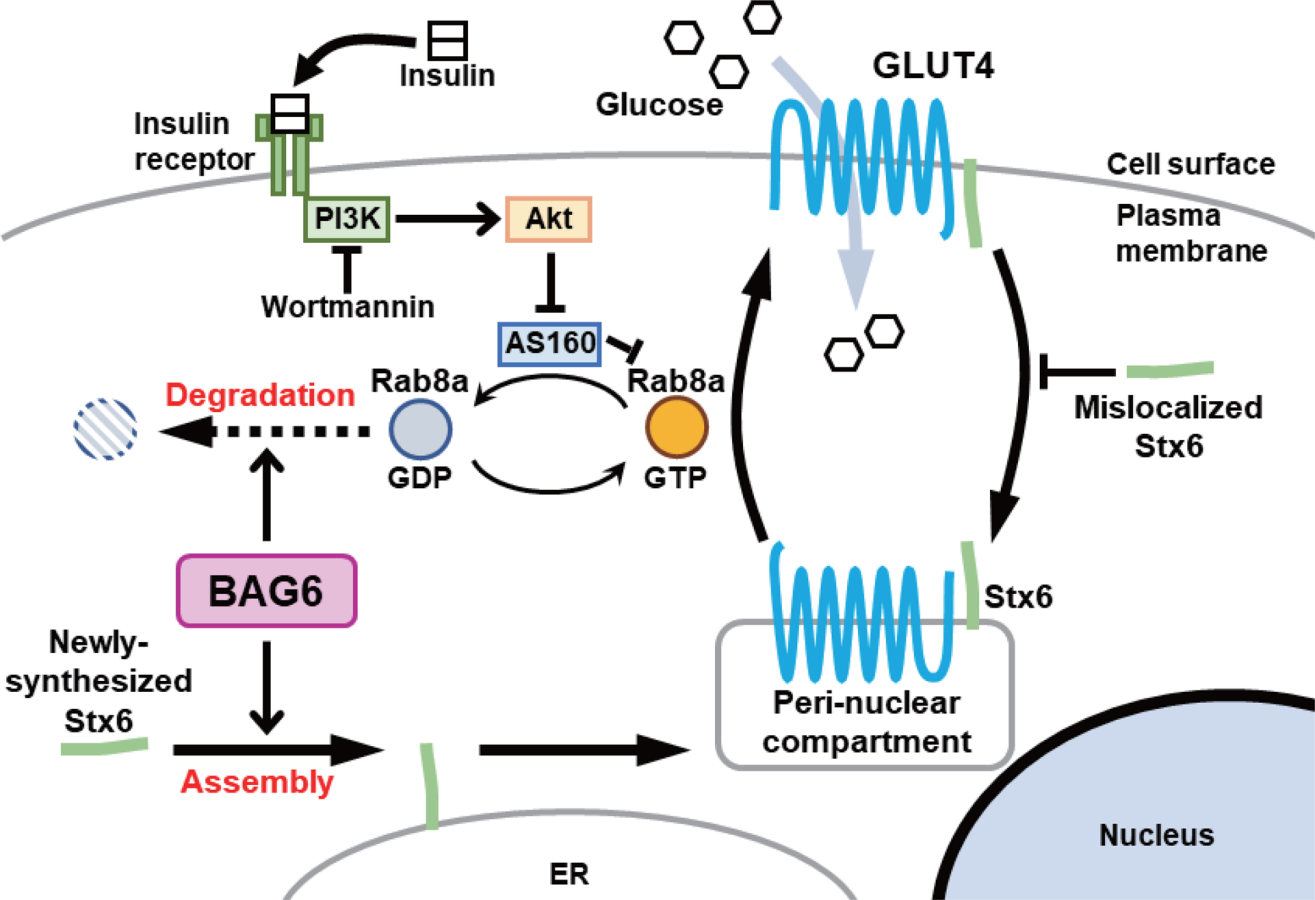
**Schematic of the possible roles of BAG6 in insulin-stimulated GLUT4 translocation.** Insulin (and downstream, PI3K-/Akt-/AS160-) -dependent translocation of GLUT4 from the peri-nuclear compartment to the cell surface depends on Rab8a small GTPase. BAG6 is critical for the degradation of the GDP-bound inactive form of Rab8a (Takahashi et al., 2019), whose accumulation impairs the function of GTP-bound Rab8a. BAG6 also plays a partly redundant role in the assembly of newly synthesized Stx6 into the ER membrane. Collectively, dysfunction of BAG6 results in accumulation of GDP-bound Rab8a, as well as cytosolically mislocalized Stx6 whose accumulation impairs the function of membrane-anchored Stx6. Therefore, defects in BAG6 lead to defective cell surface expression of GLUT4 in response to insulin, which in turn leads to reduced glucose incorporation into the cells.

The insulin-induced cell surface expression of GLUT4 is regulated by a Rab8a/PI3K-mediated membrane trafficking mechanism (Summers et al., 1998; Ishikura et al., 2007; Randhawa et al., 2008; Ishikura and Klip, 2008; Sun et al., 2010; 2014; Sadacca et al., 2013; Lim et al., 2015; Li et al., 2017) (Fig. 8). In accordance with this, we confirmed that Rab8a depletion and/or PI3K inhibition caused defects in the cell surface expression of GLUT4 (Fig. 6A,C), similar to the case for BAG6 knockdown (Fig. 4B). Because Rab8a was recently reported as a target of BAG6 for the control of membrane trafficking events (Takahashi et al., 2019), we reasoned that the observed defects in GLUT4 translocation in BAG6-suppressed cells were primarily attributable to the dysfunction of Rab8 family proteins (Fig. 8). However, BAG6 knockdown still had a residual effect on the cell surface expression of GLUT4 even in the presence of a PI3K inhibitor (Fig. 6C). Because the BAG6 complex is also known as a component of the TA protein membrane insertion machinery, which mediates the biogenesis of SNARE proteins (Mariappan et al., 2010; Leznicki et al., 2010; Kawahara et al., 2013; Kuwabara et al., 2015; Lee and Ye, 2013; Shao et al., 2017), we suspected that some of the observed trafficking defects of GLUT4 induced by BAG6 depletion might be due to the dysfunction of SNARE proteins in addition to that of Rab proteins. Therefore, we examined the potential effects of BAG6 knockdown on a SNARE TA protein, Stx6, which was reported to be associated with GLUT4 translocation (Perera et al., 2003; Shewan et al., 2003; Foley and Klip, 2014). We noticed that the membrane integration efficacy of Stx6 was partly disturbed by BAG6 depletion. Since cytosolically mislocalized syntaxin fragments cause defects in the intracellular trafficking of membrane vesicles (Perera et al., 2003), the insufficient assembly of Stx6 into the ER (and the accumulation of mislocalized Stx6 in the cytosol) might partly explain the defects in GLUT4 translocation caused by BAG6 depletion (Fig. 8). In contrast to the case for Stx6, BAG6 knockdown had little effect on the membrane integration of other TA proteins, such as RAMP4, BNIP1, Sec61β and GS28, as reported previously (Casson et al., 2017; Takahashi et al., 2019). The membrane integration defect of endogenous Stx6 observed in BAG6-suppressed cells was also partial (Fig. 7A). These observations are probably due to the existence of redundant machinery for TA protein biogenesis, such as the SRP-independent (SND) targeting pathway, which can accommodate a wide range of membrane proteins that are not fully dependent on the BAG6/transmembrane recognition complex (Aviram et al., 2016; Casson et al., 2017; Haßdenteufel et al., 2017; Shao et al., 2017). A further examination of the contributions of Stx6 and related SNARE defects is necessary to clarify how deficits in TA protein biogenesis contribute to the BAG6-induced defects in GLUT4 localization. In addition, it is probable that glucose uptake in our GLUT4-transgenic CHO cells might utilize other glucose transporters such as GLUT1 because the incorporation of a glucose analog into these cells was compromised only modestly by BAG6 depletion. The contributions of BAG6 to other glucose transporters remain to be addressed.

This study provides the first clue for a link between BAG6 and GLUT4 translocation to the plasma membrane (Fig. 8). It was recently suggested that *Bag6* is encoded within potential obesity susceptibility loci on chromosome 6p21, and a significant difference in the alternative splicing of *Bag6* transcripts was observed between overweight individuals with type 2 diabetes and lean individuals with normal glycemia (Kaminska et al., 2016). It was also reported that *Bag6* splicing is highly determined by body mass index (BMI) (Kaminska et al., 2016). Furthermore, Hager and colleagues assessed the role of genetic variation in determining protein level variation in obesity and suggested that rare polymorphisms in the *Bag6* gene might be associated with the observed changes in leptin protein expression in subjects with type 2 diabetes (Carayol et al., 2017). In many cases of obesity and type 2 diabetes, GLUT4 levels are downregulated on the surface of adipose and muscle cells and glucose transport is also impaired (Zierath et al., 1996; Bogan, 2012). In this study, we examined whether BAG6 was necessary for the cell surface expression of GLUT4, which is critical for insulin-dependent glucose incorporation. Our observations suggest that BAG6 possesses essential but seemingly multiple regulatory roles in the translocation of GLUT4 to the cell surface (Fig. 8). Because these findings highlight a link between BAG6 and glucose uptake in mammalian cells, further extensive investigations of the function and regulatory mechanism of BAG6 are necessary to clarify the etiology of type 2 diabetes and obesity, which are widespread and increasingly prevalent lifestyle-related modern diseases.

## MATERIALS AND METHODS

### Plasmid construction

Full-length cDNAs for *Glut4*, *IR* and *Stx6* were amplified by PCR from cDNA library derived from mouse C3H10T1/2 cells. The PCR fragments were cloned into pCI-neo/pCI-puro-based mammalian expression vectors (transcription was driven by a CMV promoter; Promega) with a C-terminal mCherry tag (GLUT4), C-terminal 3xT7 tag (IR), or C-terminal OPG tag with N-terminal Flag-/T7-tags (Stx6) with their products, respectively. The 3×Flag tag was inserted between Pro^66^ and Gly^67^ in the extracellular domain of GLUT4 (designated as *Flag*GLUT4-mCherry) (Kanai et al., 1993). These expression vectors were used for experiments after verification of the sequence of inserted DNA.

### Mammalian cell culture and transfection

Chinese hamster (*Cricetulus griseus*)-derived CHO-K1 cells were cultured in Ham's F-12 medium (Wako) supplemented with 10% heat-inactivated fetal bovine serum (FBS) at 37°C under 5% CO_2_ atmosphere. Transfections of the expression vectors were performed with polyethyleneimine ‘MAX’ transfection reagent (Polysciences, Inc.) or NEPA 21 Electroporator (Nepa-Gene) according to the protocols supplied by the manufacturers. At 24 h after transfection, the cells were harvested and subjected to immunological analysis unless otherwise noted.

### Stable cell line construction

Transgenic CHO-K1 cell lines stably co-expressing *Flag*GLUT4-mCherry and IR-T7 were produced by transfecting CHO-K1 cells with the respective expression vectors as follows. At 24 h after transfection with the pCI-neo-IR-T7 expression vector, 750 μg/ml G-418 (Wako) was added to the culture media. At 7 days after G-418 selection, T7-positive cells were isolated by limiting dilution. Subsequently, the T7-positive cells were transfected with the pCI-puro-*Flag*GLUT4-mCherry expression vector, and were selected with 7.5 μg/ml puromycin (Wako). At 4 days after puromycin selection, Flag- and T7-double positive cells were isolated by limiting dilution, and were used as *Flag*GLUT4-mCherry/IR-T7 transgenic CHO-K1 cells (clones 8-20 and 8-9). The stable expression of these proteins was verified by western blot analysis with anti-Flag (GLUT4) and anti-T7 (IR) antibodies. Flag-inserted GLUT4 expression was also confirmed by mCherry-derived fluorescence. IR-T7 was introduced into the cells to enhance the insulin signal, although we found that exogenous IR was dispensable for insulin-dependent Akt phosphorylation in the parental CHO-K1 cells. Therefore, we prepared *Flag*GLUT4-mCherry expressing CHO-K1 cells without IR-T7 (clone 51-25) in the same way.

### RNA interference

*Bag6* depletion in rodent CHO-K1 cells was performed as described previously (Takahashi et al., 2019) with three independent hamster-specific duplex siRNAs covering the targeted sequences: 5′-GACAUUCAGAGCCAGCGAAtt-3′ (*Bag6* siRNA#2), 5′-CCUUCAAUCUUCCUAGUGAtt-3′ (*Bag6* siRNA#3) and 5′-GACACUUCCUGAAGAGCCAtt-3′ (*Bag6* siRNA#5).

Note that the sequences above were different to the case in BAG6 knockdown in human cells (Takahashi et al., 2019).

*Rab8a* depletion in CHO-K1 cell was performed as described previously (Takahashi et al., 2019) with duplex siRNAs covering the targeted sequence: 5′-GCAUCAUGCUGGUCUACGAtt-3′ (*Rab8a* siRNA#1)

STX6 depletion in CHO-K1 cell was performed with duplex siRNAs covering the targeted sequence: 5′-GGCAAAUUGUCAGGGACAUtt-3′ (*Stx6* siRNA)

MISSION siRNA Universal Negative Control 1 (Sigma-Aldrich) was used as a general negative control in every experiment (Takahashi et al., 2019).

Transfections of CHO-K1 cells with duplex siRNA were performed using Lipofectamine 2000 (Thermo Fisher Scientific) or Lipofectamine RNAiMAX (Thermo Fisher Scientific), according to the protocols provided by the manufacturer. The efficacy of each siRNA was verified by immunoblot with their specific antibodies listed in the next section.

### Immunological analysis

For western blot analyses, whole-cell lysates were subjected to SDS-PAGE and transferred onto polyvinylidene fluoride transfer membrane (GE Healthcare). The membranes were then immunoblotted with specific antibodies as indicated and then incubated with horseradish peroxidase-conjugated antibody against mouse or rabbit immunoglobulin (GE Healthcare), followed by detection with ECL western blotting detection reagents (GE Healthcare). For Phospho-Akt (Ser^473^) blotting, we used Can Get Signaling^R^ immunoreaction enhancer solution (TOYOBO), according to the protocols provided by the manufacturer.

For glycosylation analysis of Stx6-OPG, CHO-K1 cells were washed with ice-cold phosphate-buffered saline (PBS) and lysed with immunoprecipitation (IP) buffer containing 20 mM Tris-HCl pH 7.5, 5 mM EDTA, 150 mM NaCl, 1% Nonidet P-40 and 100 µM MG-132. The lysates were sonicated, centrifuged at 20,630×***g*** for 15 min at 4°C, and mixed with 4 µl of anti-Flag M2 affinity gel (Sigma-Aldrich) for 30 min at 4°C. After the beads had been washed three times with the IP buffer, the precipitates were incubated with or without five units of the de-glycosylation enzyme: PNGase F (Promega) for 2 h at 37°C. After the beads had been washed two times with the IP buffer, the immuno-complexes were eluted by SDS sample buffer.

The following antibodies were used in this study: anti-BAG6 rabbit polyclonal (Minami et al., 2010), anti-Stx6 rabbit polyclonal (Synaptic Systems), anti-Phospho-Akt (Ser^473^) rabbit polyclonal (Cell Signaling Technology, Inc.), anti-Rab8a monoclonal (610844, BD Transduction Laboratories), anti-Flag M2 monoclonal (Sigma-Aldrich), anti-T7-tag monoclonal (Novagen), anti-calnexin polyclonal (Sigma-Aldrich), anti-α-tubulin (DM1A) monoclonal (Sigma-Aldrich), anti-β-actin polyclonal (A2066, Sigma-Aldrich), anti-mCherry polyclonal (Proteintech) antibodies.

### Microscopic cell surface GLUT4 expression assay

As a microscopic assay for the insulin-stimulated cell surface expression of GLUT4, *Flag*GLUT4-mCherry/IR-T7 transgenic CHO-K1 cells were washed twice with phosphate-buffered saline (PBS), and the cells were further incubated with Ham's F-12 medium (without serum) for 3 h. Subsequently, the cells were exposed to 1 μg/ml insulin (Wako) for 30 min as indicated. After insulin stimulation, the cells were chilled immediately on ice. The cells were fixed with 4% paraformaldehyde for 30 min on ice and treated with 50 mM glycine at 4°C for 10 min as a quenching procedure. Cells were then reacted with anti-Flag M2 monoclonal IgG as a primary antibody, following incubation with Alexa Fluor 488 goat anti-mouse IgG as a secondary antibody. Note that the plasma membrane was intentionally left non-permeabilized in this assay in order to detect cell surface GLUT4 exclusively. Cell surface immunofluorescent images were obtained using a BZ-X700 fluorescence microscope (Keyence). Cell number was measured using the Hybrid Cell Count software of BZ-Analyzer. ImageJ 1.45s (National Institutes of Health) was used for image processing. Statistical significance was calculated by the chi-square test. To observe the nucleus, cells were treated with 2.5 μg/ml Hoechst 33342.

### Flow cytometry analysis of insulin-stimulated GLUT4 exocytosis

*Flag*GLUT4-mCherry/IR-T7 transgenic cells were exposed to insulin as described in a previous section. When applicable, 100 nM wortmannin (Sigma-Aldrich) was added at 10 min before insulin treatment. After reaction with an anti-Flag M2 monoclonal antibody and Alexa Fluor 488 goat anti-mouse IgG without permeabilization, the living cells were dissociated from the plates using Accutase (Innovative Cell Technologies), and Alexa Fluor 488-derived fluorescence was measured on a flow cytometer BD Accuri™ C6 (Becton Dickinson). To calculate normalized surface levels of the GLUT4 reporter, mean surface GLUT4 reporter fluorescence (derived from Alexa Fluor 488) was divided by that of the insulin-untreated controls. Data from populations of ∼100,000 cells were analyzed using BD Accuri C6 software. Statistical significance was calculated based on experiments run in at least three biological triplicates.

### Other microscopic observations

For the observation of intracellular *Flag*GLUT4-mCherry, CHO-K1 cells (clone 51-25) were grown on micro coverglass (Matsunami), fixed by incubating in 4% paraformaldehyde for 30 min on ice. To observe the nucleus, cells were treated with 2.5 μg/ml Hoechst 33342. mCherry fluorescent images were obtained by laser scanning confocal microscopy system LSM710 (Carl Zeiss). ZEN (Black edition) was used for image processing, and ImageJ 1.45s (National Institutes of Health) was used for quantification of perinuclear GLUT4 signals and image processing.

For the immunostaining of IR-T7 and endogenous Stx6, CHO-K1 cells (WT) were grown on micro coverglass (Matsunami), fixed by incubating in 4% paraformaldehyde for 30 min on ice, and then permeabilized with 0.05% digitonin for 10 min in room temperature (RT). After permeabilization, cells were blocked with 3% FBS solution in PBS for 1 h at RT, reacted with appropriate primary antibodies as indicated at 4°C for overnight, and were subsequently reacted with secondary antibodies, Alexa Fluor 488 goat anti-rabbit IgG antibodies. Immunofluorescent images were obtained by laser scanning confocal microscopy system as described above.

### Subcellular fractionation

At 72 h after transfection with *Bag6* siRNA, the cells were harvested by centrifugation at 3000×***g***, and gently lysed using a syringe with a 27 G needle in hypotonic buffer [50 mM HEPES (pH7.4), 10 mM KCl, 1 mM DTT] on ice. The cell homogenates were centrifuged at 3000×***g*** for 2 min and subsequently ultra-centrifuged at 100,000×***g*** for 30 min at 4°C using a Beckman Coulter Optima^R^ MAX TLS-55 rotor. The resulting 100,000×***g*** supernatants were used as cytosolic fractions, while the precipitates were used as membrane-containing fractions. After extensive washing of the precipitates with PBS, the precipitates (membrane fractions) and supernatants (cytosolic fractions) were dissolved in SDS-PAGE sample buffer for western blot analysis. Successful isolation of the membrane and cytosolic fractions was verified by immunoblot analysis with anti-calnexin (a membrane fraction marker) and anti-α-tubulin (a cytoplasmic marker) antibodies.

### 2-NBDG uptake assay

CHO-K1 cells were washed twice with PBS. After incubation in Ham's F-12 medium (without serum) for 2 h, the cells were washed twice with PBS and incubated with Dulbecco's modified Eagle's medium (without serum and glucose) for 1 h. Thereafter, the cells were treated with 1 µg/ml insulin for 15 min. Glucose analog uptake was initiated by the addition of 50 µg/ml 2-deoxy-2-[(7-nitro-2,1,3-benzoxadiazol-4-yl)amino]-D-glucose (2-NBDG). Uptake was terminated by washing the cells twice with Cell-Based Assay Buffer (Cayman Chemical) at 15 min after 2-NBDG additions. The cells were harvested by trypsin-EDTA treatments. 2-NBDG uptake was measured using a BD Accuri™ C6 Flow Cytometer in all experiments, and the mean of the measurements was used for analysis. GLUT1-dependent glucose uptake was estimated using the results from 150 nM BAY-876 treatment (Siebeneicher et al., 2016). Data from populations of ∼50,000 cells were analyzed using BD Accuri C6 software. Statistical significance was calculated based on experiments run in biological triplicate.

### Statistics

Data are presented as mean±s.d., and were analyzed using a Student's *t*-test, if not stated otherwise. All analyzed experiments used at least three biological replicates to compute statistical significance. In all statistical analysis, two-tailed *P*<0.05 was considered statistically significant.

## Supplementary Material

Supplementary information
