# Supplementary material for: BAG6 contributes to glucose uptake by supporting the cell surface translocation of the glucose transporter GLUT4
Source: Biol Open. 2020 Jan 24;9(1):bio047324. doi: 10.1242/bio.047324 (PMC6994957; doi:10.1242/bio.047324)
Supplement: Supplementary information [file biolopen-9-047324-s1.pdf]

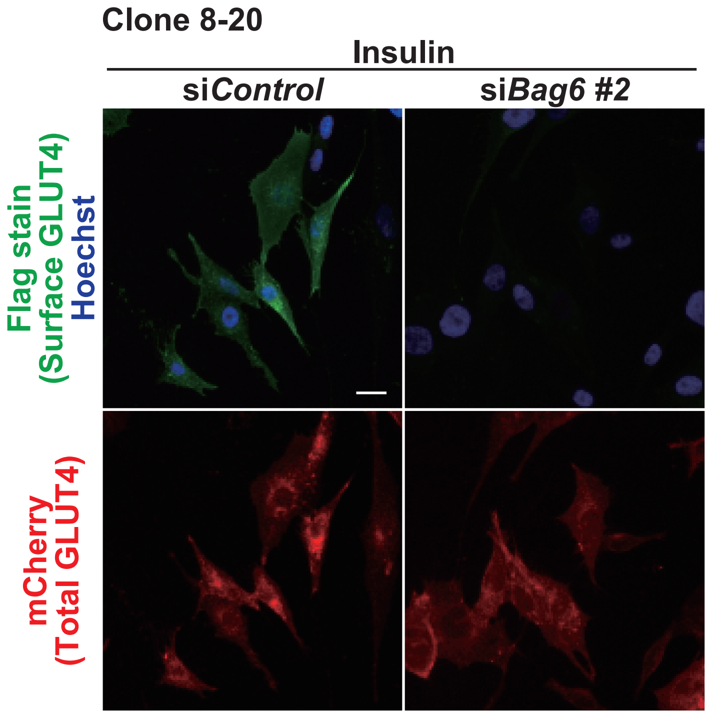

Figure S1.

In related to Fig. 3, BAG6 depletion induces defects in the cell surface expression of GLUT4 in transgenic cell line clone 8-20.

At 72 h after transfection with siRNA duplexes (5 nM each) for *cBag6* siRNA#2 (right panel) or control (left panel), plasma membrane exposed (shown as green in upper panels) or total (shown as red in lower panels) GLUT4 protein levels were observed with insulin treatment. Transgenic cell line clone 8-20 was used in this experiment. Nuclear DNA was stained with Hoechst 33342 (shown as blue). Keyence BZ-X700 fluorescence microscope was used for observations. Scale bars: 20 μm.

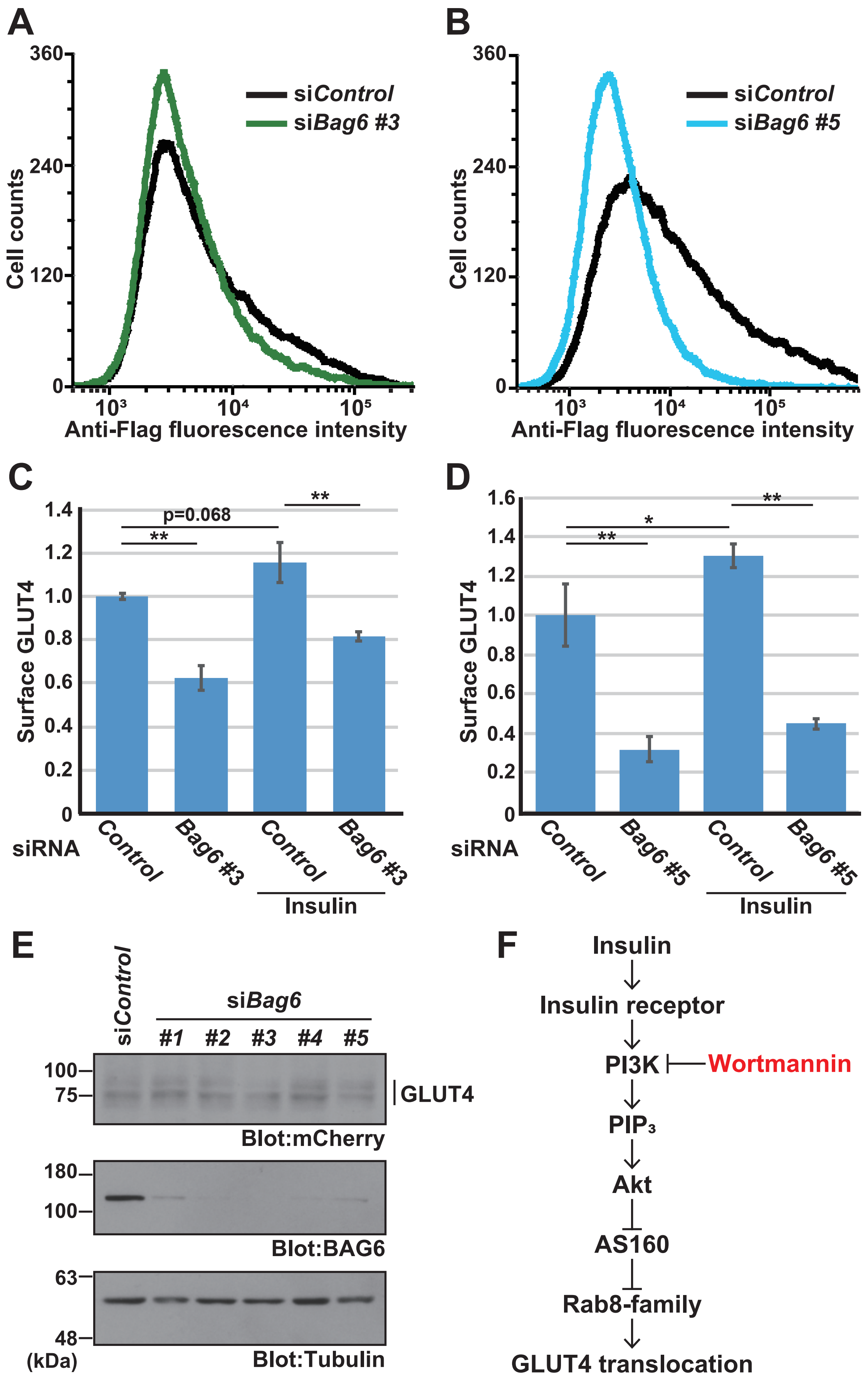

Biology Open • Supplementary information

Figure S2.

In related to Fig. 4, BAG6 knockdown reproducibly reduced the cell surface expression of GLUT4.

(A, B) Both *Bag6* siRNA #3 (A) and #5 (B) duplexes downregulated the cell surface expression of GLUT4 in non-permeabilized CHO-K1 cell line (clone 8-20). Experiments were performed as described in Fig. 4A. The live-cell flow cytometry patterns of negative control siRNA, *Bag6* siRNA#3, and *Bag6* siRNA#5 are indicated as black, green, and blue lines, respectively. Insulin (1 µg/mL) was included in the culture medium. (C,D) The independent *Bag6* siRNA #3 (C) and #5 (D) duplexes provided nearly identical effects on the cell surface expression of GLUT4. Experiments were performed as described in Fig. 4B with transgenic CHO-K1 cells clone 8-20. Statistical significance was determined by Student's *t*-test. *$p<0.05$, **$p < 0.01$. (E) Efficacy of BAG6 depletion with *Bag6* siRNA duplexes in CHO-K1 cells. Note that siRNA duplex #5 slightly down regulated total expression of *Flag*GLUT4-mCherry with unknown reason. (F) Schematic of PI3K-Akt-mediated insulin signaling pathway.

Biology Open • Supplementary information

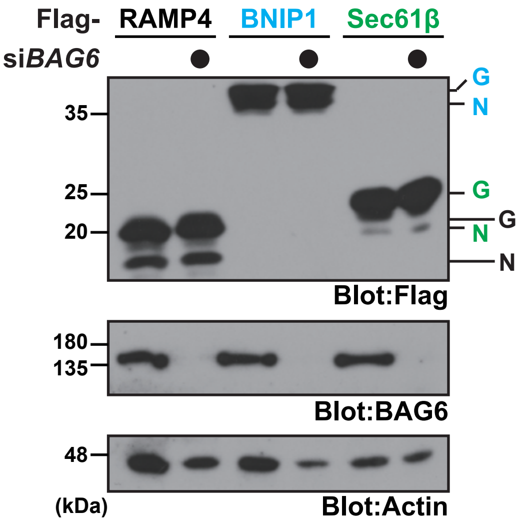

Figure S3.

In related to Fig. 7, BAG6 knockdown did not affect synthesis of major TA proteins. Glycosylation of C-terminally OPG-tagged TA proteins, RAMP4, BNIP1, and Sec61β, were not affected apparently by BAG6 knockdown in HeLa cells. Note that BAG6 depletion in human cells was performed with human-specific duplex siRNA covering the targeted sequence 5'-UUUCUCCAAGAGCAGUUUAtt-3', as described previously (Takahashi et al. 2019). Low mobility glycosylated (indicated as G) and high mobility non-glycosylated (indicated as N) signals of respective TA proteins are indicated. Actin was used as a loading control.

Biology Open • Supplementary information
